# Prevalence and infestation dynamics of intestinal protozoa infections (cryptosporidiosis, giardiasis and eimeriosis) among calves in dairy farms of Kazakhstan

**DOI:** 10.3389/fvets.2025.1635456

**Published:** 2025-10-03

**Authors:** Altay Ussenbayev, Dariyash Kurenkeyeva, Ali Aydin, Laura Sakhariya, Saidulla Ruzmatov

**Affiliations:** ^1^Veterinary Medicine Department, S Seifullin Kazakh Agro Technical Research University, Astana, Kazakhstan; ^2^Department of Information and Communication Technologies, Tashenev University, Shymkent, Kazakhstan; ^3^Department of Food Hygiene and Technology, Istanbul University-Cerrahpaşa, Istanbul, Türkiye

**Keywords:** central and northern Kazakhstan, dairy farms, calves, *Giardia*, *Cryptosporidium*, *Eimeria*, prevalence

## Abstract

**Introduction:**

This study investigates the epidemiological characteristics of significant gastrointestinal protozoa diseases among calves, including cryptosporidiosis, giardiasis, and eimeriosis, in Kazakhstan.

**Methods:**

Using cross-sectional surveys and laboratory diagnostics we examined the age and seasonal dynamics of intestinal parasitic infestations in calves, specifically focusing on Giardia spp., Cryptosporidium spp., and Eimeria spp. For this purpose, samples of feces were individually collected from 1,586 calves of varying ages and breeds in 12 industrialized farms from 11 districts of Akmola and Northern Kazakhstan Regions. Parasites were identified using Fuelleborn, Heine and ZnSO4 flotation microscopic techniques. Calves were categorized into four age groups (1–30, 31–90, 91–120, and >120 days) to evaluate the prevalence and age- associated risk of infection with above parasite species, and their combinations. Logistic regression was performed to estimate odds ratios (ORs) and 95% confidence intervals (CIs), using calves aged 1-30 days as the reference group. To examine the effects of age and time of year on the likelihood of parasitic infection ANOVA and a generalized additive logistic model (GAM) were used. It was found that in all investigated farms the age significantly influences the likelihood of infection with each parasite.

**Results and discussion:**

Cryptosporidium spp. infections were highly concentrated in the youngest calves, with prevalence 49.2% detected in the 1-30-day group. The risk of infection dropped dramatically with older age (*p* < 0.001). Prevalence Eimeria spp. in the 1-month group was 2.0% and significantly increased with age. Calves aged 31-90 days had 27.3 times higher odds of infection (95% CI: 17.07–45.35, *p* < 0.001), with elevated odds persisting in older groups (*p* < 0.001). 5.2% of the youngest calves were infected with Giardia spp., and the species was more evenly distributed across age groups, with no statistically significant variation. No significant seasonal variation in infection rates was found. These findings suggest that at the conditions of intensive dairy farming in central and northern Kazakhstan the age-targeted parasite control strategies may be more effective than seasonal approaches for managing parasitic infections' control in calves.

## Introduction

Dairy cattle farming is a strategic sector of Kazakhstan's agricultural production. As of January 1, 2025, the cattle population amounted to 7,842.6 thousand animals. Of this total, 45% were kept in household farms, 44.4% in peasant or private farms and individual enterprises, and 10.6% in industrial agricultural enterprises (1). The dairy population in Kazakhstan includes over 1.2 million cows, raised on more than 3,415 milk production farms. Over the past decade, the number of dairy farms has increased by more than 7.5 times (2).

It is worth noting that a significant share of the country's milk production is concentrated in large industrialized farms, whose numbers are steadily increasing due to ongoing urbanization. Currently, the greatest progress in the intensive dairy cattle farming development is observed in the central and northern regions of the country, owing to favorable climatic conditions and sufficient feed resources (3). This trend of intensification has led to a consistent annual import of high-breeding cattle from North America, Western and Eastern Europe to these regions, which poses a potential risk of introducing new infectious diseases to local herds from abroad (4).

Raising healthy calves is a vital condition for sustainable dairy farming, as there is a constant need to replenish and renew the dairy herd. Parasitic and infectious gastrointestinal diseases in young cattle remain one of the most significant obstacles to improving animal productivity and welfare (5). Neonatal calves are often infected with protozoa such as *Giardia* spp., *Cryptosporidium* spp., and *Eimeria* spp., which invade the intestinal epithelium and cause endemic outbreaks of parasitic diarrhea (6–10).

These parasites are transmitted via the fecal-oral route through direct or indirect contact with contaminated materials or infection sources (11). Epidemiological surveillance and case-control studies have shown that cattle can be a source for *Cryptosporidium* and *Giardia* species and genotypes that are infectious to humans, establishing this animal as zoonotic reservoir. The distribution of these pathogens and the extent of zoonotic transmission vary across different geographical regions worldwide (12–14).

Moreover, intestinal zoonoses caused by various genotypes of *Cryptosporidium* spp. and *Giardia* spp. are a growing concern for public health. The prevalence of cryptosporidiosis in humans is reported at 4.3% in developed countries and 10.4% in developing nations, with some regions experiencing infection rates as high as 69.6%. In developing countries, cryptosporidiosis is associated with growth retardation, cognitive impairment in children, and even colorectal cancer in infected individuals. Each year, cryptosporidiosis accounts for a global loss of 13 million disability-adjusted life years (DALYs) (13). The prevalence of giardiasis in humans in developing countries reaches 33%, and besides diarrhea, it is associated with arthritis and irritable bowel syndrome (14–16). Overall, outbreaks of these diseases are increasingly emerging in developed communities and remain a leading cause of child mortality in developing countries (16). Thus, understanding the factors influencing infection risks in livestock is critical for developing effective control strategies for these infestations to not only improve animal health and reduce economic loss, but also limit the public health burden (6–16).

Over the past 10 years, many studies have demonstrated a high-level infestation of young cattle with *Eimeria* spp. and significant economic losses caused by eimeriosis in dairy farms over the world (17–23). For instance, an epidemiological surveillance in Italy recorded a prevalence rate of 91.7% for *Eimeria* infections among cattle (18). In Germany, field research found that *Eimeria* spp. were present in 48.5% of fecal samples from young calves (19). In Brazil's Paraná state, *Eimeria* spp. were detected in 51.3% of samples collected from 400 calves, with mixed-species infections identified in the majority of cases (71%) (20). In Thailand's Khon Kaen region, the *Eimeria* spp. prevalence among cattle under 1 year age was 35.1%, according to a molecular study in 2020–2021 (21). A meta-analysis conducted in China showed an average infestation rate in dairy herds by eimeriosis consistently maintained at 25% (22). *Eimeria* oocysts were found in 72.4% of the total samples, with prevalence ranging from 68 to 100% across all investigated cattle farms in Ethiopia (23).

In Central Asian countries where livestock farming is crucial to the economic wellbeing of rural populations research of cryptosporidiosis, giardiasis, and other intestinal protozoan infections among productive cattle remains limited or virtually nonexistent. Nevertheless, few studies indicate that *Cryptosporidium* spp. and *Giardia intestinalis* are widely distributed among farm animals in this region. For example, cryptosporidiosis was identified in 22.7% of cattle farms in the Akmola Region, predominantly in large-scale industrial enterprises, where conditions are more conducive to sustaining the biotic potential for *Cryptosporidium* spp. (24). In the Central Kazakhstan, *Cryptosporidium parvum* was detected in 35.9% of dairy farms (25). Similar data were obtained in Kyrgyzstan, where the prevalence of cryptosporidiosis and giardiasis in domestic animals demonstrated the high levels (26).

From a public health and veterinary perspective, a key aspect of controlling these parasitic infections lies in understanding their sources and transmission pathways across different geographic zones. Although cattle are recognized as a potential reservoir for human infections, the species and genotypes of *Cryptosporidium* and *Giardia* in cattle, and the extent of their zoonotic transmission in Central Asia and Kazakhstan, are still poorly understood. So, despite the rapid development of industrial dairy cattle farming in central and northern Kazakhstan, data on the prevalence of enteropathogenic protozoan infections in calves at the dairy farms in these regions remain virtually nonexistent. This highlights the urgent need for population-level research on the transmission dynamics of these parasites in regions with intensive farming, in order to improve prevention and control strategies for cryptosporidiosis and giardiasis in both animals and humans (24–26).

This study aims to assess the prevalence of gastrointestinal protozoa pathogens *Giardia* spp., *Cryptosporidium* spp., and *Eimeria* spp. in calves and examine the age-related and seasonal dynamics of infections at the industrial dairy farms located in central and northern Kazakhstan.

## Materials and methods

The cross-sectional studies were conducted in 2018–2019 and 2021–2024 through field expedition trips to the intensive dairy farms of central and northern Kazakhstan and laboratory investigations at the Parasitological Laboratory named after Professor Kadyrov, Seifullin Kazakh Agrotechnical Research University.

In Central and Northern Kazakhstan, the West Siberian climatic zone prevails with cold (minimum temperature −57.2°C) and long winters (up to 6 months) and short, often hot summers (maximum temperature +42°C). The average precipitation is from 300 to 450 mm (27). The natural and economic features of the regions, which are favorable to produce sufficient forage and grain crops, have contributed to the concentration of the main enterprises of the country's industrialized dairy cattle farming.

The large farms where the research was conducted use an intensive production system and have a closed reproduction cycle, which involves housing animals free from birth to culling in boxes. The technology provides for automatized milking, mechanized feeding and manure removal systems. Waste is processed through closed manure storage lagoons, which contributes to compliance with sanitary and environmental standards in accordance with local regulations. Feeding is based on the use of silage, haylage, concentrates and mineral additives that maintain high productivity of herds (2, 3).

**Figure 1:**
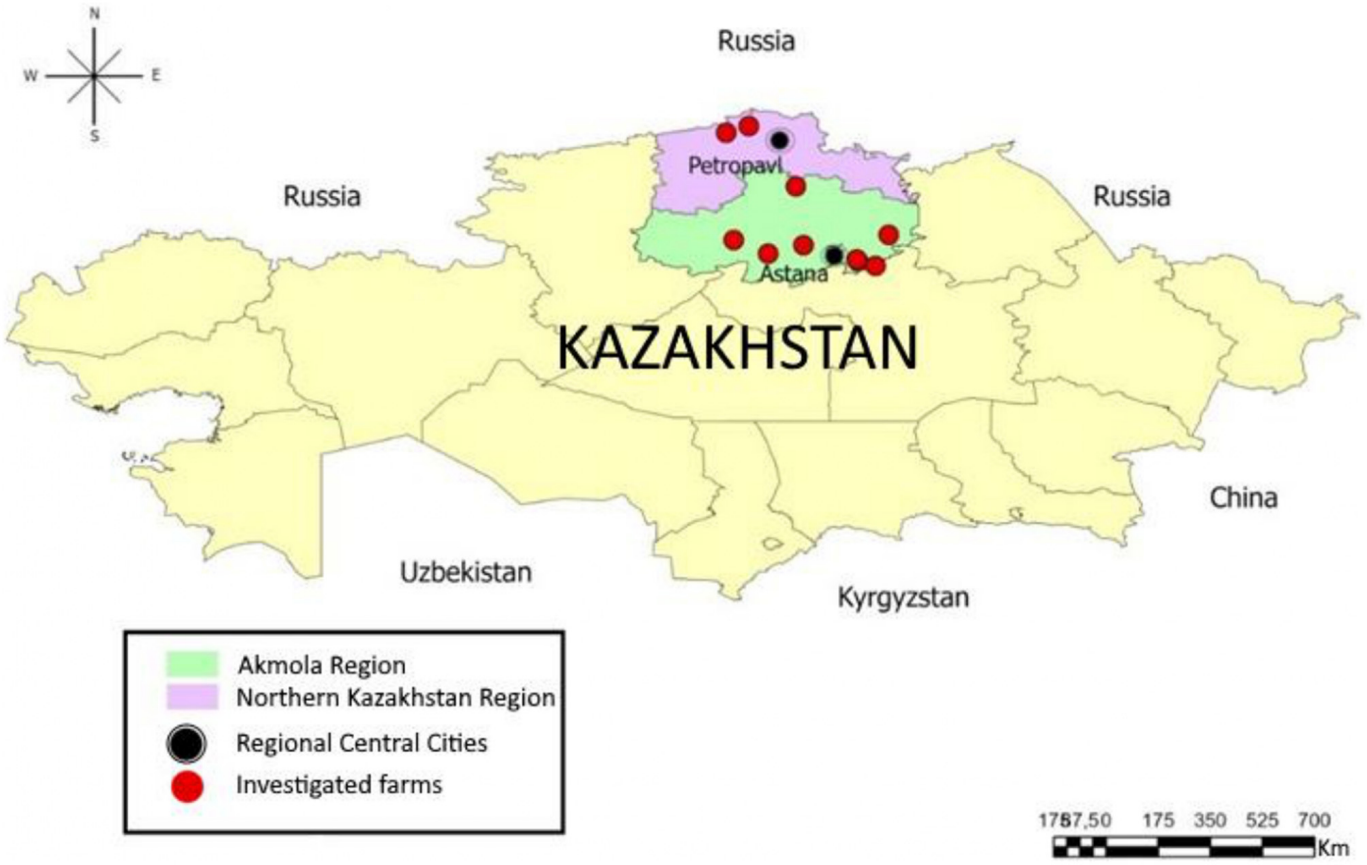
Geographical location of investigated farms.

During the field expeditions we used the stratified random sampling (28) and the population of calves in all dairy farms was categorized into age groups (1–30, 31–90, 91–120, and >120 days). A total of 1,586 feces samples were collected from calves on 12 industrialized dairy farms out of 48 milk production cattle enterprises (2) located in 11 districts of the Akmola and Northern Kazakhstan regions (Figure 1). The farms were selected for their similarity in housing, management, and feeding technologies. The study population included 12,416 calves under 1 year of age, with the number of animals per farm ranging from 475 to 1,685. The sampling strategy aimed for a representation exceeding 10% within each age group, subject to the availability of animals in those groups (Table 1).

**Table 1:** The sampling calves' number from dairy farms in Akmola and Northern-Kazakhstan regions.

**Farms no**	**District**	**Region**	**Breed**	**Number of animals under the 1-year age**	
				**Total**	**Investigated**
1	Arshaly	Akmola	Holstein	1,336	115
2	Shortandy	Akmola	Black Spotted	1,685	89
3	Atbasar	Akmola	Red Steppe	580	96
4	Burabay	Akmola	Holstein	475	90
5	Burabay	Akmola	Holstein	780	82
6	Astrakhan	Akmola	Simmental	2,166	112
7	Esil	Akmola	Simmental	1,050	92
8	Egindikol	Akmola	Red Steppe	486	73
9	Yereymentau	Akmola	Red Steppe	785	104
10	Tselinograd	Akmola	Holstein	2,450	223
11	Mamlyut	Northern-Kazakhstan	Simmental	1,161	176
12	Kyzylzhar	Northern-Kazakhstan	Simmental	512	334
					1,586

Fecal samples were collected from each animal per rectum in plastic containers, labeled and delivered immediately in the portable cooler box for laboratory analysis. Fecal samples were processed and examined microscopically to identify the presence of parasitic infections. Bauer (29), Heine (30), and flotation with zinc sulfate solution (31) techniques were used for quantifying cysts of *Eimeria* spp., *Cryptosporidium* spp., and *Giardia* spp. oocysts. Each sample was examined using three methods on a separate glass slide, identification the parasite genera was based on detection of morphologically clearly distinguishable cysts and trophozoites of *Giardia*, as well as oocysts of *Cryptosporidium* and *Eimeria*.

Data, including the calf's age, breed and gender, were recorded at the time of sample collection. Age was calculated based on the calf birthdate regarding the recording documents in each farm and animals were grouped into the above age categories. This classification allowed for an analysis of the relationship between age and level of parasitic infection of animals.

## Statistical analysis

The statistical analyses were performed using the R-4.4.0 for Windows software. A one-way analysis of variance (ANOVA) was conducted to assess whether the mean age of the sampled animals differed significantly across months. The *F*-test was used to determine the overall significance of these differences. The data on age distribution across the months sampling were visualized using boxplots, with the average age for each month represented.

For evaluating the prevalence and age-associated risk of infection with *Giardia spp*., *Cryptosporidium* spp., *Eimeria* spp., and their combinations logistic regression was performed to estimate odds ratios (ORs) and 95% confidence intervals (CIs), using calves aged 1–30 days as the reference group.

To examine the effects of age and time of year on the likelihood of parasitic infection, a generalized additive logistic model (GAM) was used. The model was fitted using the mgcv package in R. The response variable for the GAM was the presence or absence of each parasite (*Giardia, Cryptosporidium, Eimeria*), thus three models where used, one each per parasite species. The predictors in the model were the calf's age (modeled using regression splines with quantile-based knots) and the month of the year (modeled with cyclic penalized B-splines and twelve equidistant knots per month). The model was restricted to calves aged 80 days or younger due to limited samples from older calves.

The smooth functions from the GAM were visualized to display the multiplicative effects of age and season on the likelihood of infection, with transformations applied to simplify interpretation and visualization on the infection proportion scale. Statistical significance was assessed using a *p*-value threshold of < 0.05 for all analyses.

## Results

**Figure 2:**
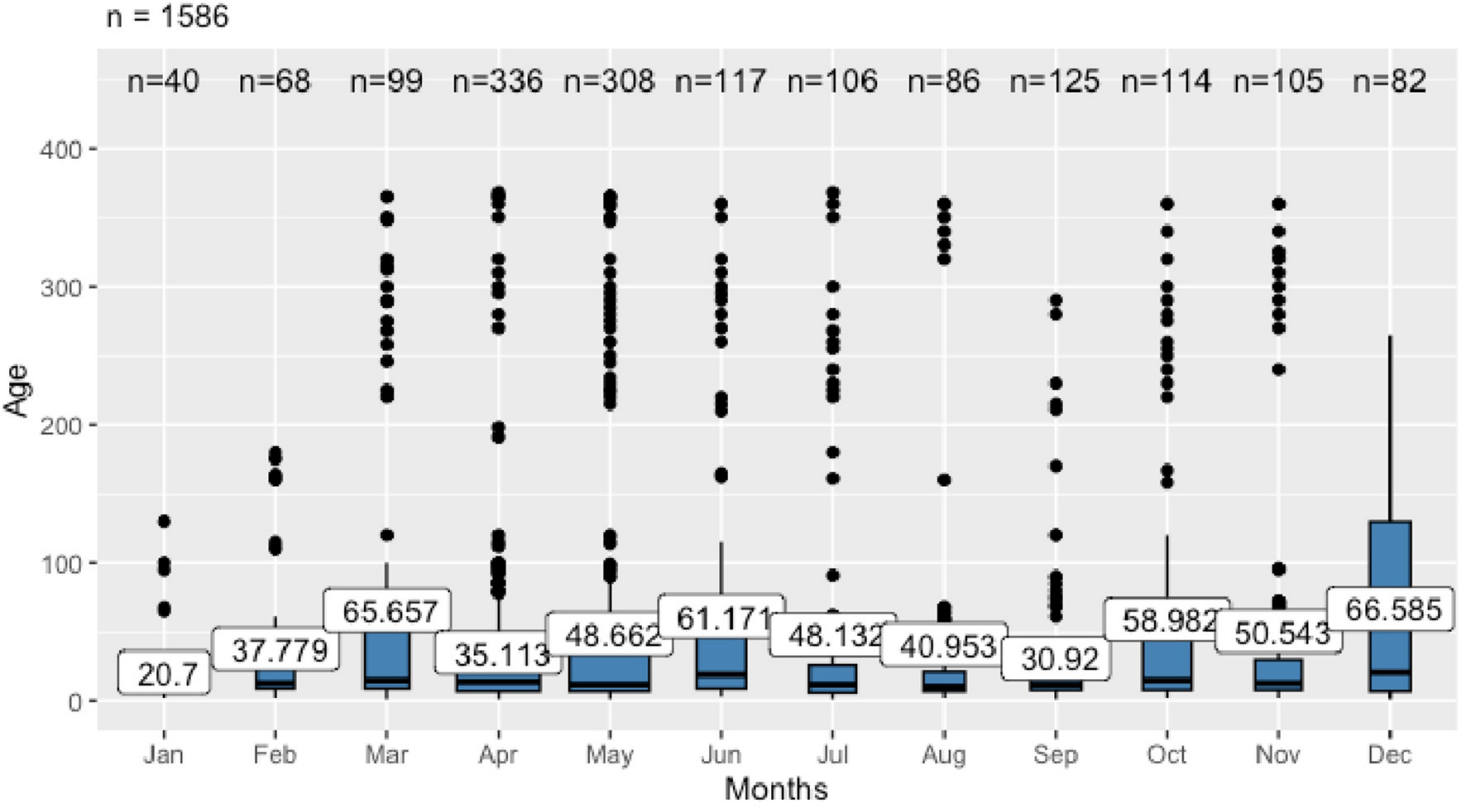
The age distribution and mean of sampled animals' age across the months (*n* = 1,586)

The ANOVA revealed a statistically significant difference in mean age among months [F_(11, 1574)_ = 3.21, *p* = 0.00025], indicating that age distribution changes over time (Figure 2). *Post-hoc* comparisons using Tukey's HSD test identified that animals sampled in April were significantly younger than those sampled in March. Other month-to-month differences were not statistically significant. The youngest calves were primarily investigated in January (mean age of 20.7 days) and September (30.9 days), while the oldest were sampled in March (65.7 days) and December (66.6 days).

The analysis of the 1,586 excrement samples revealed the presence of *Giardia* spp., *Cryptosporidium* spp., and *Eimeria* spp. in a substantial proportion of the samples. The distribution of mono- and mixed-infestations with intestinal protozoa species was presented by mono infection with all observed parasites and three associations containing two species (Table 2).

**Table 2:** The logistic regression assessment the prevalence of mono- and mixed-infestations of gut parasites among calves.

**Protozoa genera and mixed infestations**	**Calves' age in days**													
	**1–30**, ***n*** = **1,127**	**31–90**, ***n** =* **251**				**91–120**, ***n*** = **42**				>**120**, ***n*** = **166**				**Total**, ***n*** = **1,586**
	**Prv** ^*^	**Prv** ^*^	**OR**	**95%CI**	***p***	**Prv** ^*^	**OR**	**95%CI**	***p***	**Prv** ^*^	**OR**	**95%CI**	***p***	**Prv** ^*^
*Giardia* spp.	59/5.2	17/6.8	1.32	[0.73–2.25]	0.336	4/9.5	1.91	[0.56–4.94]	0.235	8/4.8	0.92	[0.40–1.85]	0.822	88/5.5
*Cryptosporidium* spp.	555/49.2	2/0.8	0.008	[0.014–0.026]	< 0.001	0				0				557/35.1
*Eimeria* spp.	23/2.0	91/36.3	27.3	[17.07–45.35]	< 0.001	14/33.3	24	[11.04–51.29]	0.001	39/23.5	14.74	[8.60–25.81]	0.001	167/10.5
*Giardia* spp.*+ Eimeria* spp.	24/2.1	29/11.6	6.00	[3.44–10.59]	< 0.001	3/7.1	3.54	[0.82–10.68]	≤ 0.046	24/14.5	7.77	[4.28–14.09	< 0.001	80/5.0
*Giardia* spp.*+ Cryptosporidium* spp.	7/0.6	11/4.4	7.33	[2.86–20.11]	< 0.001	0				0				18/1.1
*Cryptosporidium* spp. *+ Eimeria* spp.	12/1.1	4/1.6	1.50	[0.42–4.36]	0.482	0				0				16/1.0
Total	680/60.3	154/61.4				21/50.0				71/42.8				926/58.4

^*^Prv- prevalence: in the numerator – absolute number in the denominator – percentage.

It was found that *Cryptosporidium spp*. infections were highly concentrated among the youngest calves, with prevalence 49.2% detected in the 1–30 day group. The risk of infection dropped dramatically with age (31–90 days: OR = 0.0083, 95% CI: 0.0014–0.026, *p* < 0.001), and no cases were recorded in calves older than 90 days, indicating a strong age-dependence (Table 2).

In contrast, *Eimeria spp*. infections significantly increased with age. Calves aged 31–90 days had 27.3 times higher odds of infection (95% CI: 17.07–45.35, *p* < 0.001), with elevated odds persisting in older groups (91–120 days: OR = 24.0, *p* < 0.001; >120 days: OR = 14.74, *p* < 0.001) (Table 2).

*Giardia spp*. was more evenly distributed across age groups, with no statistically significant variation. Odds ratios ranged from 0.92 to 1.91 compared to the youngest group (*p* > 0.2 for all comparisons) (Table 2).

Mixed infections followed the patterns of their component parasites. Notably, *Giardia* + *Eimeria* co-infections showed significantly higher odds in calves > 120 days (OR = 7.77, 95% CI: 4.28–14.09, *p* < 0.001), consistent with the delayed rise of *Eimeria*. Meanwhile, *Giardia* + *Cryptosporidium* and *Cryptosporidium* + *Eimeria* combinations were rare or absent beyond 90 days. Some results returned undefined or extremely wide CIs (e.g., OR = 0 with CI upper bounds exceeding 1e+69), reflecting sparse data and zero-case in older age groups (Table 2).

**Figure 3:**
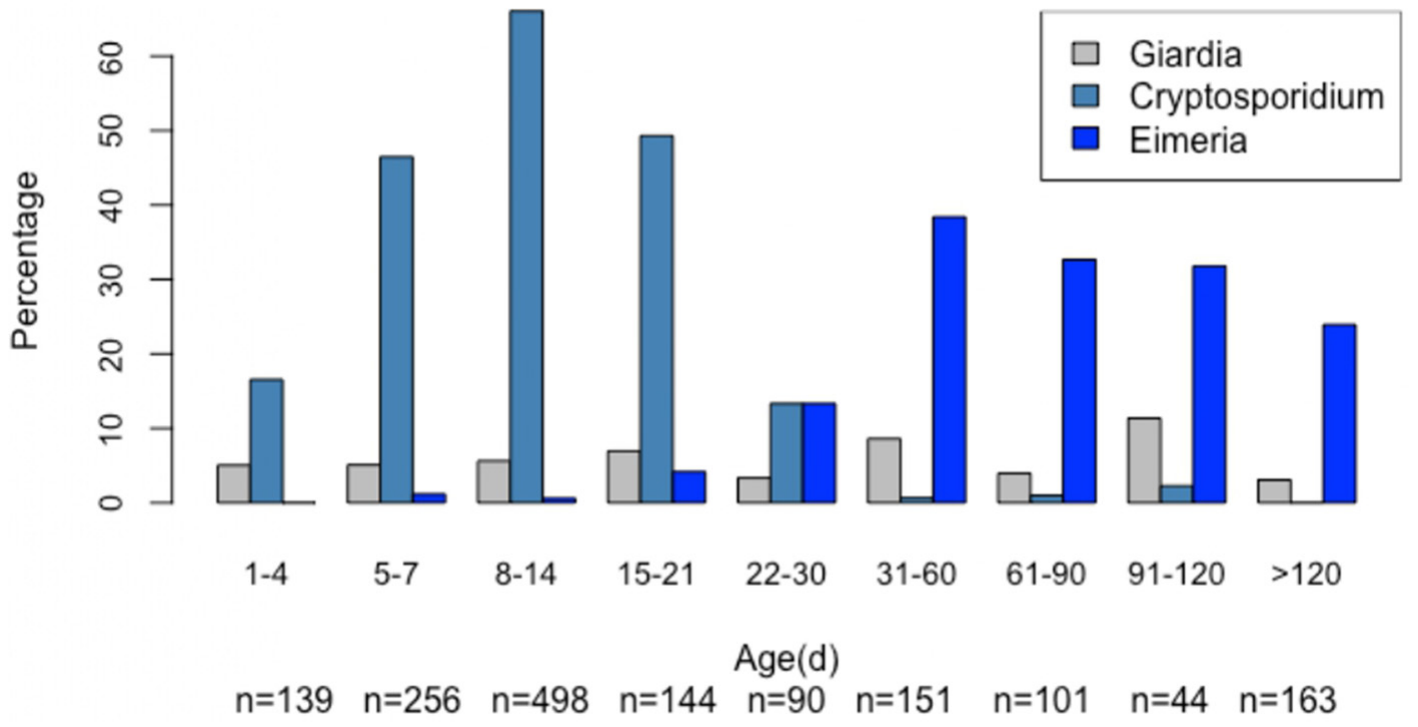
Age-related frequencies of *Giardia, Cryptosporidium*, and *Eimeria* cysts/oocysts (*n* = 1,586).

*Giardia* cysts were found in 88 out of 1,586 samples (5.55%). The *Giardia*-positive calves ranged in age from 3 to 290 days (mean = 35.73 days, median = 11.5 days), with the highest cyst excretion rate (11.36%) observed in calves aged 91–120 days (Figure 3).

**Figure 4:**
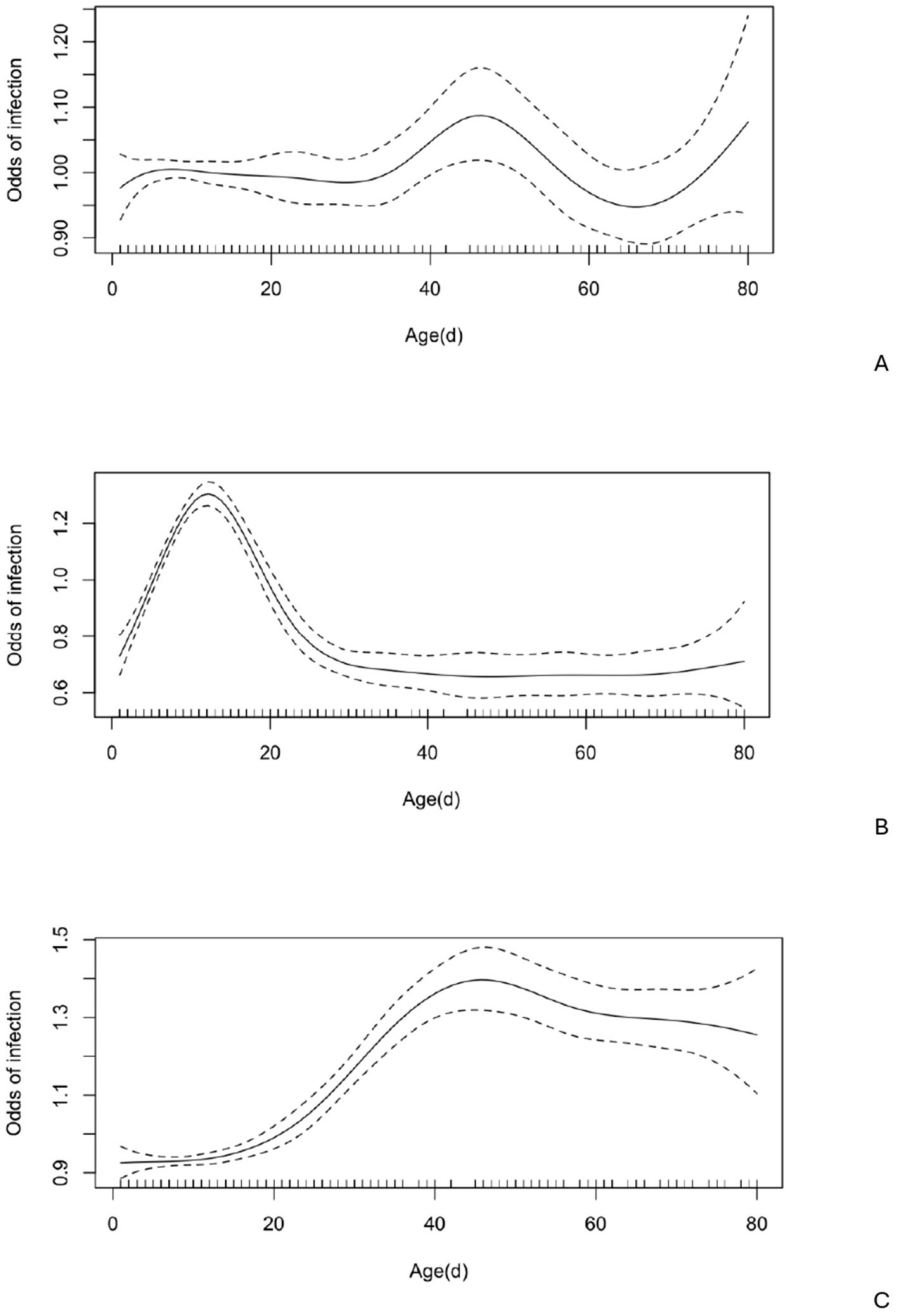
Odds of infection at various ages (*n* = 1,347) with 95% confidence intervals. A. *Giardia* spp.; B. *Cryptosporidium* spp.; C. *Eimeria* spp.

Notably, age had a nonlinear effect on the likelihood of *Giardia* spp. infection, with the odds of infection gradually increasing by 1.14 times from approximately 35–47 days, then rising sharply between 70 and 80 days (Figure 3). There was no seasonal effect (Figure 4) on *Giardia* infection (*p* > 0.05).

*Cryptosporidium* oocysts were detected in 557 out of 1,586 samples (35.12%). These oocysts exhibited the size and morphology typical of *C. parvum*. The *Cryptosporidium*-positive calves ranged in age from 1 to 91 days (mean = 10.84 days, median = 10 days), with the highest oocyst excretion rate (66.06%) observed in calves aged 8–14 days (Figure 3).

**Figure 5:**
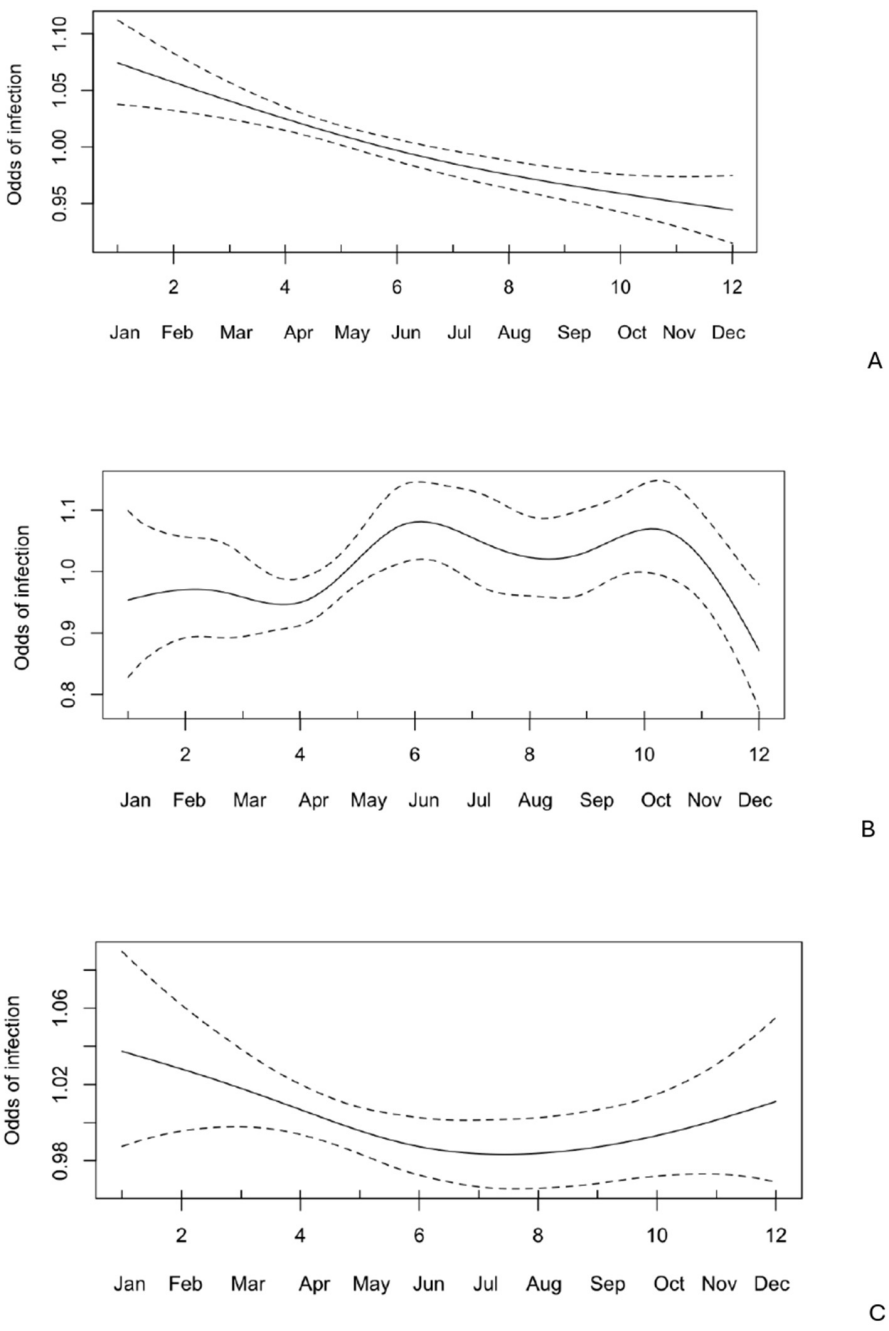
Odds of infection across different months (*n* = 1,347) with 95% confidence intervals for *Giardia* spp., *Cryptosporidium* spp., and *Eimeria* spp.

Notably, age had a nonlinear effect on the likelihood of *Cryptosporidium spp*. infection, with the odds of infection increasing by up to 1.36 times until the calves reached 12 days old. After this, the odds gradually decreased, approaching zero around 30 days of age (Figure 4). No significant seasonal variation (Figure 5) in *Cryptosporidium* infection was observed (*p* > 0.05).

*Eimeria* oocysts were found in 168 out of 1,586 examined samples (10.59%). The *Eimeria*-positive calves ranged in age from 7 to 360 days (mean = 103 days, median = 62 days), with the highest oocyst excretion rate (38.41%) observed in calves aged 31–60 days (Figure 3). Notably, age had a nonlinear effect on the likelihood of *Eimeria spp*. infection, with the odds increasing steadily by up to 1.48 times from approximately 14 to 45 days (Figure 4).

There was no seasonal effect (Figure 5) on *Eimeria* infection (*p* > 0.05).

## Discussion

According to the present study conducted at the intensive dairy farms in northern and central Kazakhstan, the prevalence of *Giardia spp*. in cattle under 1 year age was 5.5%, which is significantly lower compared to data from many previous studies in other world regions. Overall, *Giardia* is widely distributed among cattle globally, with prevalence ranging from 9 to 73% of animals at the herd level. At the last decade the parasite was detected in 45–100% of farms in European countries (7, 11, 14, 32). In Brazil, up to 40% of dairy cows may carry *Giardia intestinalis*, often in mixed infections with *Cryptosporidium* spp. (12). In China, the prevalence of *G. intestinalis* in calves reaches 33.7%, correlating with high rates of diarrhea and reduced weight gain (33). A systematic review reports that in Europe the prevalence of *G. intestinalis* in cattle ranges from 10 to 35% with higher rates observed in intensive farming systems and in neonatal animals (39.6%) (15). Infected animals often show signs of chronic diarrhea, reduced productivity, impaired nutrient absorption, and, as a result, significant economic losses (34). The comparably low prevalence of calve giardiasis in this research could be explained by a strong continental climate of central and northern Kazakhstan with average temperature higher 25–30°C in May-July, what influenced to the indoor environmental housing systems of farms. Such conditions decreased the viability of cysts and served for limitation the population dynamics of *Giardia* spp. (35).

In our study, the highest detection rate of *Giardia spp*. was observed in calves aged 61–90 days. This finding aligns with other research indicating that calves under 4 months of age are most commonly infected. This is often explained by the weak immune status of young animals (34). Previous studies have reported peak *Giardia* infections at 4–7 weeks and 4–5 months of age (13). In a study in Germany, a commercially available molecular tests showed that 51.2% of calves aged 2–16 weeks were infected with *Giardia* spp. (36).

Our analysis suggests that *Giardia* infection in cattle is not seasonally dependent, likely due to the closed-cycle nature of industrial dairy farming, which maintains stable hygiene and environmental conditions. This observation is supported by similar conclusions from other studies (37–43). It was also established that a prior infection with *Cryptosporidium* spp. may enhance the pathogenic potential of *Giardia* spp. (44).

*Cryptosporidium* spp. was the most commonly detected pathogen in calves from dairy farms in our study, with a prevalence of 35.1%. Most infected animals were under 30 days of age, suggesting infection with *C. parvum*, the species most frequently associated with young calves, although infection with another species cannot be completely ruled out. Previous PCR studies on dairy cattle in the North Kazakhstan have identified *C. parvum* and *C. andersoni* as a prevalent species, and *C. parvum* was particularly significant in young calves, while *C. andersoni* was more common in older animals. These findings highlight the importance of understanding the prevalence and transmission of *Cryptosporidium* species in dairy farming for both animal health and potential zoonotic risks (45). In Germany, the prevalence of *C. parvum* in calves ranges from 21.5 to 44.0%, which is comparable to the results of this study (46). Globally, prevalence rates of *Cryptosporidium spp*. in calves range from 6.2 to 100% (44). The age-specific distribution in our findings, with the highest detection rate (66.7%) in calves aged 8–14 days, is consistent with previous research (48). Furthermore, the absence of seasonal variation in the risk of *Cryptosporidium* infection also aligns with findings from other studies (44–48).

Oocysts of *Eimeria* spp. were found in 10.5% of calves at the intensive dairy farms in central and northern Kazakhstan. Compared to Austria and Colombia, where the prevalence of *E. bovis* and *E. zuernii* in calves under 1 year of age is 59.4 and 75.5%, respectively (49, 50), the observed rate is relatively low. As species differentiation was not performed in this study, it is presumed that most oocysts detected likely belonged to the pathogenic species described above (50). Recent epidemiological studies show that the prevalence of coccidiosis in calves and young cattle varies depending on climate, housing system, and the effectiveness of preventive measures (51). For example, in Brazil, the infection rate of calves in intensive dairy farms reaches up to 80%, while it is lower—around 45%—in pasture-based systems (52). In China, the prevalence of *Eimeria* spp. in calves ranges from 55 to 70%, with higher risks in regions with high humidity (53). The low eimeriosis infestation found in this study is likely a result of the extremely continental climate in the research areas (27). The parasite's life cycle is dependent on environmental conditions, and the long, cold seasons (roughly 6 months) would inhibit the sporulation (22) and survival of the infectious stage due to persistently low temperatures in the housing utilities.

According to our findings, the highest prevalence of Eimeria infections was observed in calves aged 61–90 days. This agrees with other literature reporting the highest infection risk in animals aged 3–12 months (17, 51).

Thus, the results of this study highlight the significant role that age plays in the likelihood of parasitic infections in calves. *Giardia* and *Cryptosporidium* infections were more prevalent in younger calves, while *Eimeria* infections were most common in calves aged 31–60 days. This pattern suggests that as calves grow and their immune systems develop, they become less susceptible to certain parasitic infections, particularly *Giardia* and *Cryptosporidium*. However, *Eimeria* infection appears to be associated with exposure to contaminated environments, which increases as calves begin feeding with dry feed. So, protozoa infections in calves displayed distinct age-related patterns. *Cryptosporidium spp*. poses a major threat to newborn animals for the first 3 weeks, while *Eimeria* spp. became prominent after the first month of life. *Giardia* spp. exhibited a more stable prevalence across age groups.

Interestingly, no significant seasonal variation in infection rates was observed for any of the three parasites. This suggests that other factors, such as age and immune status, may play a more crucial role in infection risk than seasonal changes in temperature or humidity. These findings challenge the traditional focus on seasonal parasite management and suggest that age-targeted control strategies could be more effective in intensive dairy farming systems. Therefore, we propose to continue the study of these parasite infections in fattening and small household cattle farming systems in the here investigated regions. To enhance parasite infection surveillance, future studies will be incorporated molecular diagnostic techniques like PCR to overcome limitations in current research, specifically the lack of species-level identification. This will improve the accuracy and reliability of prevalence estimates and allow for better tracking of species-specific infections.

## Conclusion

This study of dairy herds in intensified farms of central and northern Kazakhstan emphasizes the importance of age in determining the prevalence of parasitic infections in calves. The findings suggest that *Giardia* and *Cryptosporidium* infections are most common in very young calves, while *Eimeria* infections peak in calves aged 31–60 days. Age-targeted parasite control strategies including the treatment with specific antiparasitic drugs are likely to be more effective than seasonal approaches in the industrialized farms of Kazakhstan, and further research should focus on optimizing these strategies. Additionally, understanding the immune development of calves and the factors that influence susceptibility to parasitic infections will be critical for improving the calf health and welfare as well as productivity in the livestock industry.

## Data Availability

The original contributions presented in the study are included in the article/supplementary material, further inquiries can be directed to the corresponding author.
